# Association of Interleukin-6 Signalling with the Muscle Stem Cell Response Following Muscle-Lengthening Contractions in Humans

**DOI:** 10.1371/journal.pone.0006027

**Published:** 2009-06-24

**Authors:** Bryon R. McKay, Michael De Lisio, Adam P. W. Johnston, Ciara E. O'Reilly, Stuart M. Phillips, Mark A. Tarnopolsky, Gianni Parise

**Affiliations:** 1 Department of Kinesiology, McMaster University, Hamilton, Ontario, Canada; 2 Department of Pediatrics, McMaster University, Hamilton, Ontario, Canada; 3 Department of Medical Physics & Applied Radiation Sciences, McMaster University, Hamilton, Ontario, Canada; University of Birmingham, United Kingdom

## Abstract

**Background:**

The regulation of muscle stem cells in humans in response to muscle injury remains largely undefined. Recently, interleukin-6 (IL-6) has been implicated in muscle stem cell (satellite cell)-mediated muscle hypertrophy in animals; however, the role of IL-6 in the satellite cell (SC) response following muscle-lengthening contractions in humans has not been studied.

**Methodology/Principal Findings:**

Eight subjects (age 22±1 y; 79±8 kg) performed 300 maximal unilateral lengthening contractions (3.14 rad.s^−1^) of the knee extensors. Blood and muscle samples were collected before and at 4, 24, 72, and 120 hours post intervention. *IL-6*, IL-6 receptor (*IL-6Rα*), *cyclin D1*, *suppressor of cytokine signling-3* (*SOCS3*) mRNA were measured using quantitative RT-PCR and serum IL-6 protein was measured using an ELISA kit. JAK2 and STAT3 phosphorylated and total protein was measured using western blotting techniques. Immunohistochemical analysis of muscle cross-sections was performed for the quantification of SCs (Pax7^+^ cells) as well as the expression of phosphorylated STAT3, IL-6, IL-6Rα, and PCNA across all time-points. The SC response, as defined by an amplification of Pax7^+^ cells, was rapid, increasing by 24 h and peaking 72 h following the intervention. Muscle *IL-6* mRNA increased following the intervention, which correlated strongly (R^2^ = 0.89, p<0.002) with an increase in serum IL-6 concentration. SC IL-6Rα protein was expressed on the fiber, but was also localized to the SC, and IL-6^+^ SC increased rapidly following muscle-lengthening contractions and returned to basal levels by 72 h post-intervention, demonstrating an acute temporal expression of IL-6 with SC. Phosphorylated STAT3 was evident in SCs 4 h after lengthening contraction, and the downstream genes, *cyclin D1* and *SOCS3* were significantly elevated 24 hours after the intervention.

**Conclusions/Significance:**

The increased expression of STAT3 responsive genes and expression of IL-6 within SCs demonstrate that IL-6/STAT3 signaling occurred in SCs, correlating with an increase in SC proliferation, evidenced by increased Pax7^+^/PCNA^+^ cell number in the early stages of the time-course. Collectively, these data illustrate that IL-6 is an important signaling molecule associated with the SC response to acute muscle-lengthening contractions in humans.

## Introduction

Muscle specific stem cells, named satellite cells (SCs), are necessary for muscle repair and regeneration and are known to reside in skeletal muscle between the basal lamina and the sarcolemma. Successful repair of muscle damage is dependent on SCs to activate, proliferate and terminally differentiate [1], [2]. The contribution of new myonuclei to muscle fibers, from SCs, is necessary to promote postnatal muscle growth and to prevent the loss of functional capacity and increased morbidity and mortality associated with muscle loss in advanced age and disease (bedrest, cachexia, etc.) [3]–[5]. The paired box transcription factor, Pax7, is necessary for the induction of the myogenic response following muscle injury by promoting the transcription of the myogenic regulatory factors (MRFs) as well as the maintenance of the SC compartment through self-renewal [6]–[11]. It has been previously shown that the SC pool is responsive to exercise-induced muscle damage [12]–[14]; however, the regulation of this response remains poorly understood. Many growth factors and cytokines such as, hepatocyte growth factor (HGF), insulin-like growth factor-one (IGF-1), IL-4 and leukemia inhibitory factor (LIF), among many others, have been implicated in playing some role in regulating the SC response [15]–[20]. However, the contribution of many of these potential SC regulators to hypertrophy and muscle repair, *in vivo*, is not known.

IL-6 is a multifunctional cytokine primarily involved in immune function [21]; however, the chronic systemic elevation of IL-6 in disease states has been associated with promoting a catabolic state leading to muscle atrophy [22]–[24]. It has been previously demonstrated that exercise can induce increases in muscle derived *IL-6* mRNA and plasma IL-6 concentrations following exercise [25], [26]. However, whether changes in IL-6 concentration can influence human muscle SC function has yet to be examined.

Several recent studies provide key evidence that IL-6 signaling may be important in myogenisis. It has been shown that skeletal muscle possesses the IL-6 receptor (IL-6Rα) on the sarcolemma and that the IL-6Rα is responsive to exercise [27]. It is well established that IL-6 is produced by muscle in response to inflammation and exercise [28]–[31] (for review see ref. [26]). However, it was only recently that IL-6 was shown to play a significant role in SC-mediated muscle hypertrophy [17]. IL-6 knock-out (IL-6^−/−^) mice demonstrated a blunted hypertrophic response and less SC-related myonuclear accretion compared to wild-type mice following compensatory hypertrophy [17]. Furthermore, SCs from IL-6^−/−^ mice demonstrated an impaired proliferative capacity, both *in vivo* and *in vitro*, which was shown to be related to a lack of IL-6 mediated signal transducer and activator of transcription-3 (STAT3) signaling [17]. Although IL-6 signaling has been shown to play an important role in the hypertrophic response to overload in mice, the role of IL-6 in muscle repair following injury in humans has not been elucidated.

Using a model of repeated muscle-lengthening contractions, which we have previously shown to induce significant muscle damage [32]–[34], we sought to induce a SC response and examine the association of IL-6 with the SC response. We hypothesized that IL-6 would play a key role in the human SC-mediated response to contraction-induced muscle damage. Furthermore, based on the work of Serrrano et al., [17] we hypothesized that SC-mediated regulation by IL-6 would act through STAT3 signaling.

## Results

### The IL-6 Response to Muscle-Lengthening Contractions (MLC)

**Figure 1:**
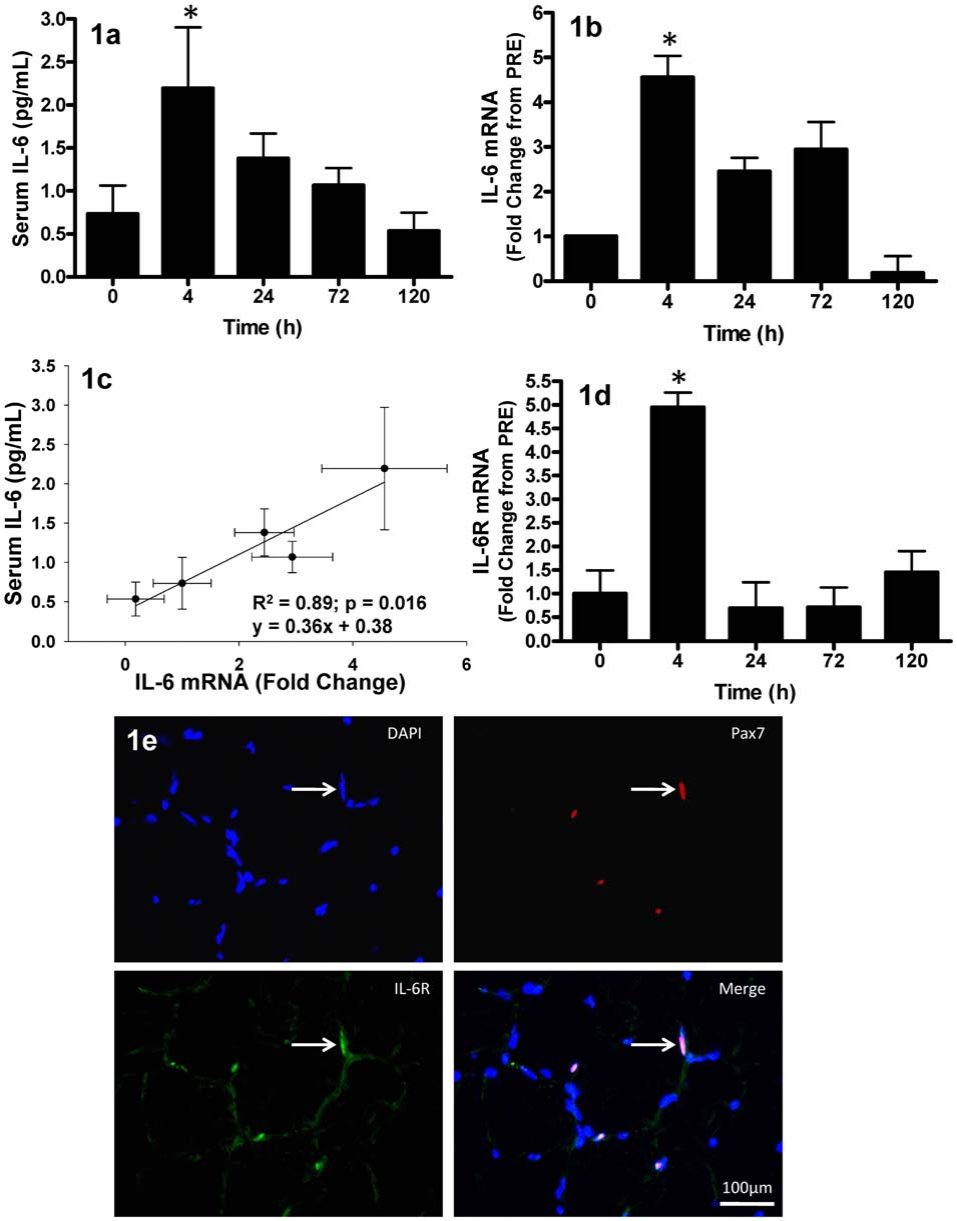
Circulating IL-6 and muscle IL-6 mRNA and IL-6 receptor response muscle lengthening contractions (MLC). (1a) Average serum interleukin-6 (IL-6) concentration. Time 0 hr corresponds to pre-intervention values; all other time-points correspond to post-intervention time (hr). (1b) Relative *IL-6* mRNA expression, expressed as fold-change from 0 hr. (1c) Pearson correlation of serum IL-6 concentration versus muscle *IL-6* mRNA (fold-change), correlation is representative of the individual data points and presented as mean values (•)±SD (error bars). (1d) Relative IL-6 receptor (*IL-6Rα*) mRNA expression, expressed as fold-change from 0 hr. (1e) Immunofluorescent image (40×) of a muscle cross-section triple-stained for Pax7 (red), IL-6Rα (green) and nuclei (DAPI = blue); IL-6Rα staining is apparent on the sarcolemma and satellite cell membrane. White arrow denotes one of the Pax7^+^ nuclei which co-localized with IL-6Rα (scale bar = 100 µm). Values are reported as mean±S.E.M. Mean values represent the mean for all 8 subjects per time-point (8 samples per time-point, 40 samples total). *p<0.05 vs. 0 hr.

In response to 300 maximal muscle-lengthening contractions, serum IL-6 protein increased by approximately 2-fold (0.75 pg/mL PRE to 2.25 pg/mL at T4, p<0.05) by T4 with respect to baseline (PRE) levels. However, this response was transient, returning to PRE levels by T120 (Fig. 1a). Interestingly, muscle *IL-6* gene expression demonstrated a parallel response. *IL-6* mRNA increased 4.5-fold from PRE at T4 (p = 0.019) and returned to baseline levels by T120 (Fig. 1b). Serum IL-6 was strongly correlated to the increase in muscle *IL-6* mRNA (r^2^ = 0.89, p = 0.016) (Fig. 1c).

In order to test whether *IL-6Rα* gene expression was responsive to our MLC protocol, we measured muscle *IL-6Rα* mRNA. *IL-6Rα* gene expression was significantly increased in whole muscle at T4 (p<0.001); however, these changes were transient as expression levels returned to PRE values by T24 (Fig. 1d). In addition, it has been recently shown that primary myoblasts isolated from mice possess the IL-6Rα, however it remains unknown whether human satellite cells possess the IL-6Rα [17]. Our observational immunofluorescent analysis revealed that Pax7^+^ cells co-localized with IL-6Rα, verifying that SCs in humans possess the IL-6Rα (Fig. 1e).

### Satellite Cell Response

**Figure 2:**
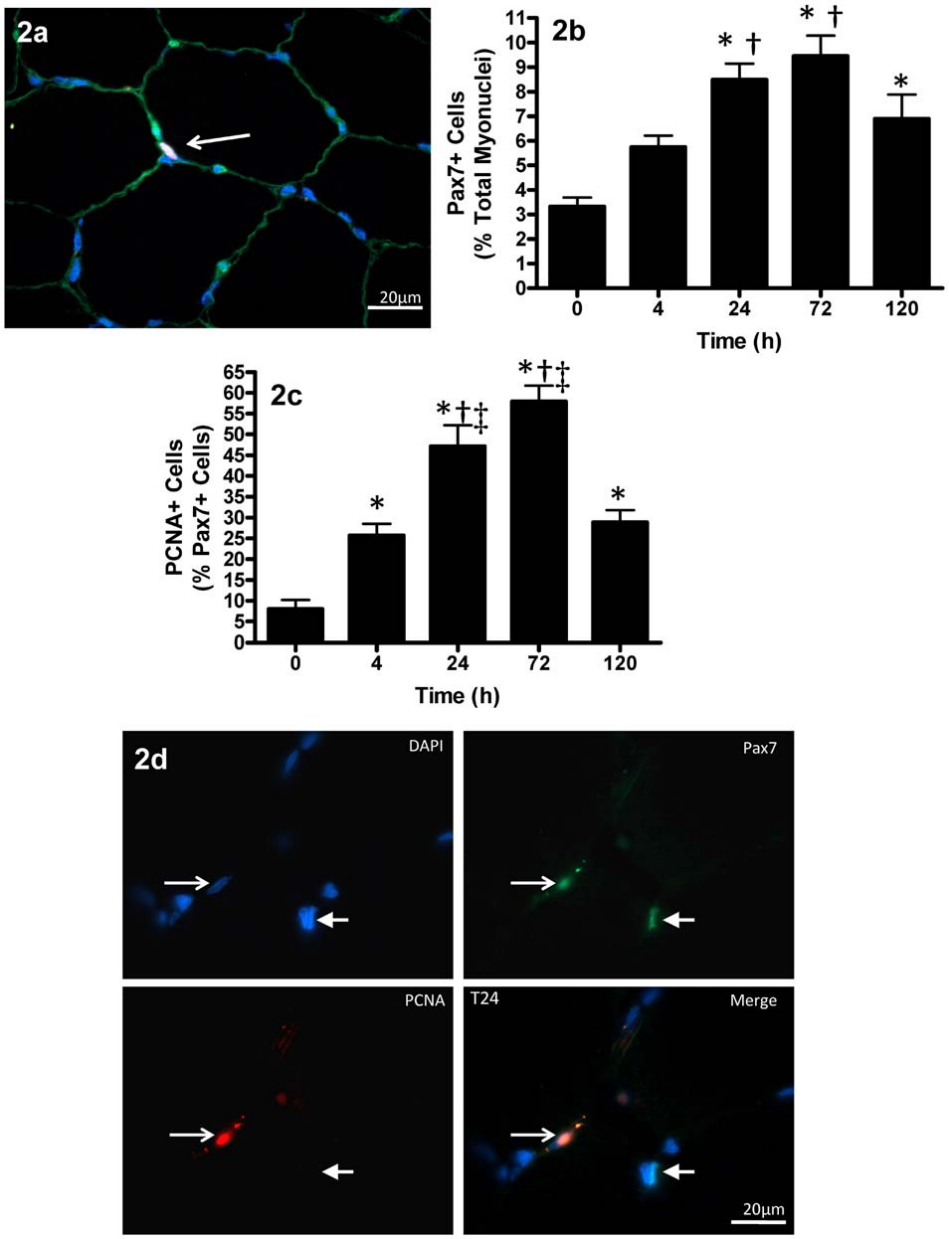
Muscle satellite cell response to muscle lengthening contractions (MLC). (2a) Triple-immunofluorescent staining of 7 µm muscle cross-section for satellite cells (red = Pax7^+^), laminin (green) and nuclei (DAPI = blue). Thin arrow denotes the Pax7^+^ cell located beneath the basal lamina in the satellite cell niche (scale bar = 20 µm). (2b) Pax7^+^ nuclei expressed as a percentage to total myonuclei following MLC. (2c) PCNA^+^ cells expressed as a percentage of all Pax7^+^ satellite cells following MLC. (2d) Triple-immunofluorescent staining of 7 µm muscle cross-section for satellite cells (green = Pax7^+^), PCNA (red) and nuclei (DAPI = blue). Thin arrow denotes the PCNA^+^/Pax7^+^ cell, thick arrow denotes a Pax7^+^ nuclei with no PCNA staining (scale bar = 20 µm). Values are reported as mean±S.E.M. Mean values represent the mean for all 8 subjects per time-point (8 samples per time-point, 40 samples total). *p<0.05 vs. 0 hr; †p<0.05 vs. 4 hr; ‡p<0.05 vs. 120 hr.

The response of Pax7^+^ cells to contraction-induced injury was quantified across the post intervention time-course (see supplemental Fig. S1). The number of Pax7^+^ cells (expressed as a percentage of total myonuclei) increased 155% at T24 (3.32±3.37% PRE to 8.48±0.66% T24), peaking at T72 with a 184% increase from PRE (3.32±3.37% PRE to 9.45±0.83% T72; p<0.005). The number of Pax7^+^ cells began to decrease at T120, but remained elevated as compared to PRE (p = 0.007) (Fig. 2a, b).

To determine the percentage of Pax7^+^ cells that were proliferating, muscle cross-sections were co-stained with an antibody against proliferating cell nuclear antigen (PCNA), a marker for cells in early G1 and S phase of the cell cycle (see Fig. S3). Pax7/PCNA co-staining revealed an increase in Pax7^+^/PCNA^+^ cells as early as T4 (26% of Pax7^+^ cells were PCNA^+^), peaking at T72 (58% of Pax7^+^ cells were PCNA^+^) and remaining elevated at T120 (Fig. 2c, d). In order to test whether IL-6 was present in SCs, we conducted triple immunofluorescent staining for Pax7, IL-6, and DAPI.

**Figure 3:**
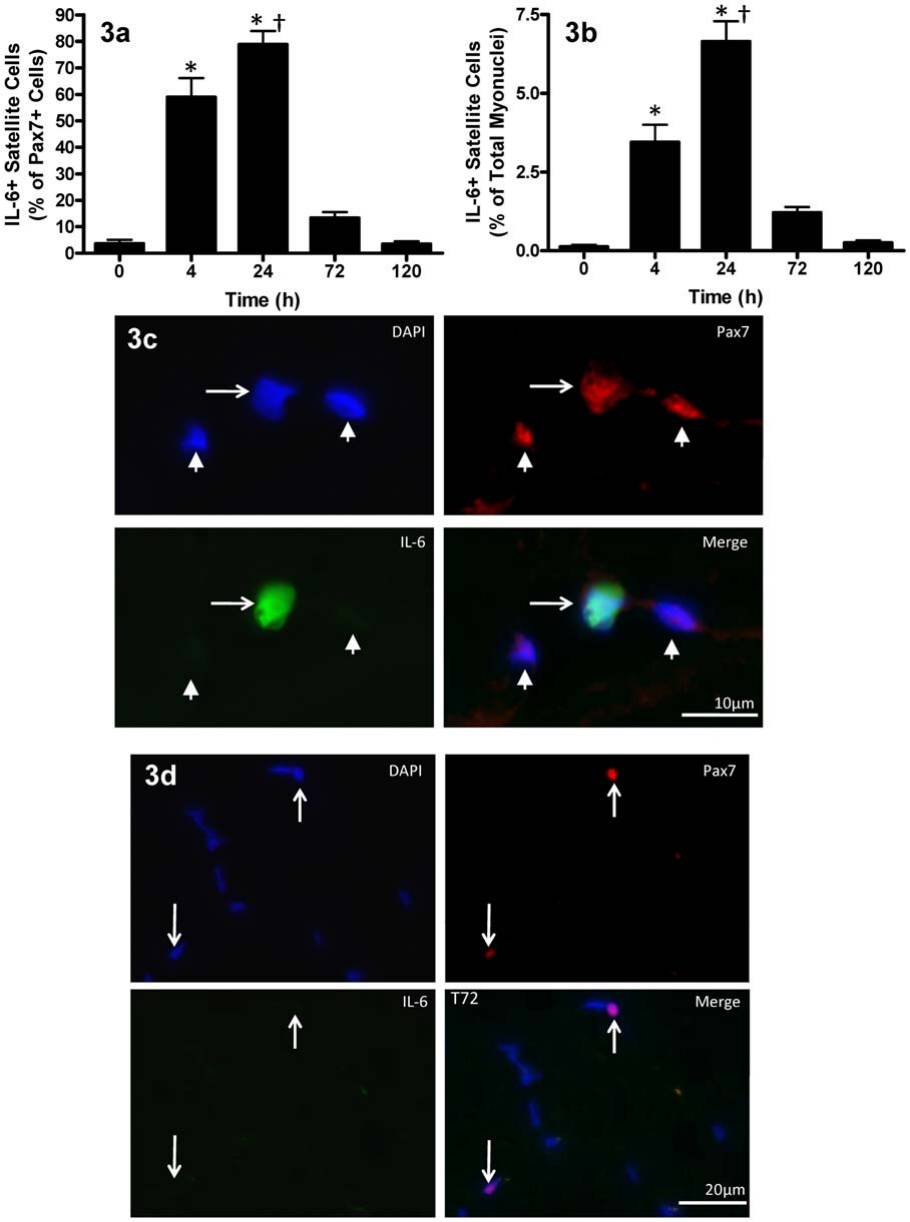
Satellite cell IL-6 protein expression following muscle lengthening contractions (MLC). (3a) Il-6^+^ satellite cells expressed as a percentage of all Pax7^+^ satellite cells following MLC. (3b) Il-6^+^ satellite cells expressed as a percentage of total myonuclei following MLC. Values are reported as mean±S.E.M. Mean values represent the mean for all 8 subjects per time-point (8 samples per time-point, 40 samples total). *p<0.05 vs. 0 hr; †p<0.05 vs. 4 hr. (3c)IL-6/Pax7 co-localization following muscle lengthening contractions (MLC). Triple-immunofluorescent staining of 7 µm muscle cross-sections for satellite cells (red = Pax7^+^), IL-6 protein (green) and nuclei (DAPI = blue). The thin arrow denotes a Pax7^+^/IL-6^+^ cell and the thick arrows denote Pax7^+^ cells with no IL-6 staining (scale bar = 10 µm). (3d) Representative image of 72 hr (T72) where white arrows denotes a Pax7^+^ cells with no IL-6 staining (scale bar = 20 µm).

Immunofluorescent triple staining of Pax7, IL-6, and DAPI, revealed a substantial increase in the localization of IL-6 protein within SCs (see Fig. S2). Figure 3a and 3b demonstrate the time-course of IL-6 protein expression in Pax7^+^ cells. IL-6^+^ SCs (expressed as a percentage of all Pax7^+^ cells) increased from ∼2.16±1.47% of satellite cells PRE, to 58.94±7.2% at T4 (Fig. 3c; p<0.001) and peaked at 80.7±5.9% of satellite cells at T24 (p<0.001). By T72 the number of IL-6^+^ SCs dropped 83% from T24 (Fig. 3d) and returned to baseline levels by T120. The increase in Pax7^+^/IL-6^+^ cells positively correlated with the increase in Pax7^+^/PCNA^+^ cells observed from PRE to T24 (R^2^ = 0.52, p<0.0001); however, after T24 there was no correlation as SCs no longer expressed IL-6, whereas PCNA^+^ SC number was still increasing.

### JAK2/STAT3 signaling

**Figure 4:**
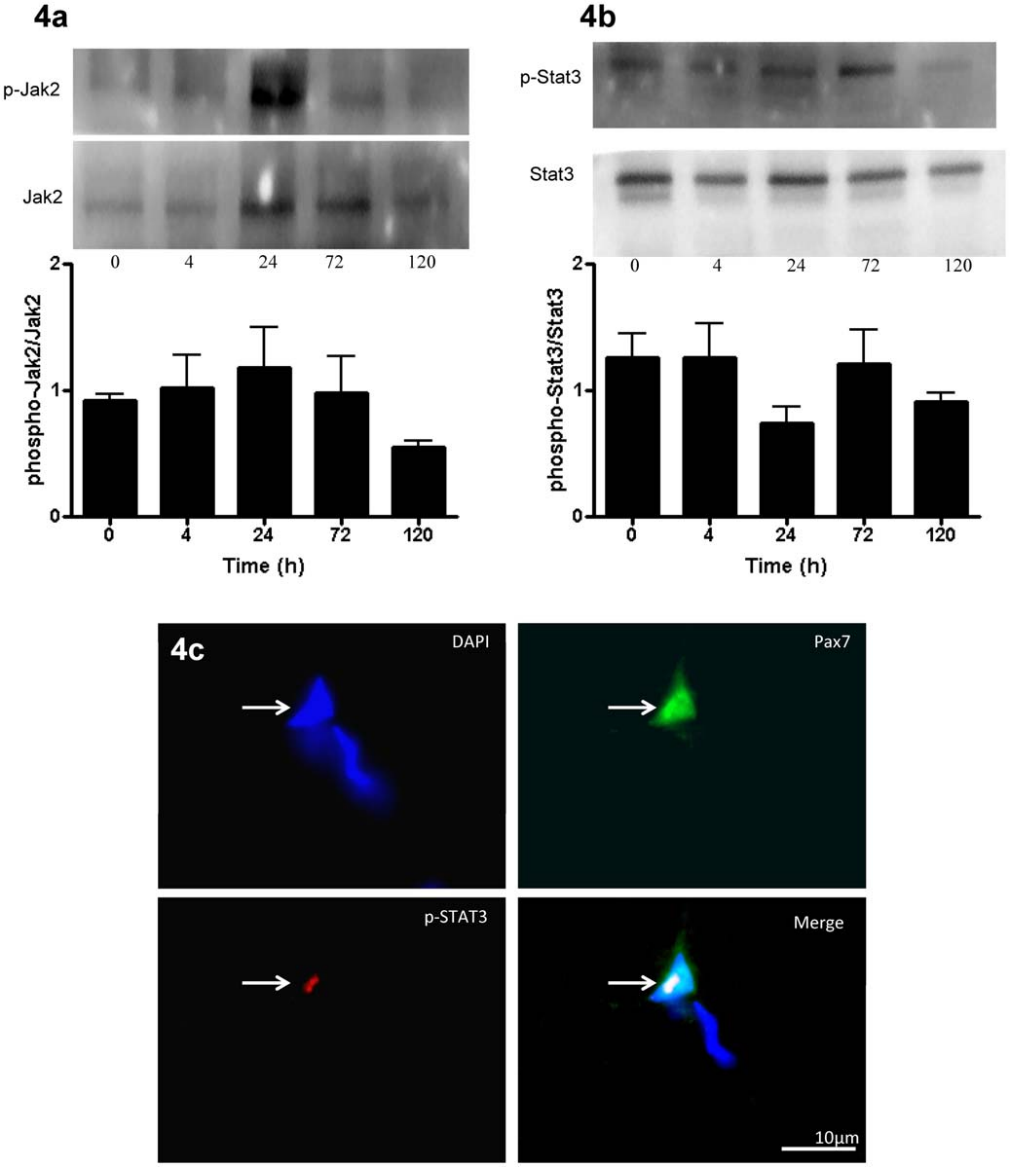
JAK2/STAT3 activity following muscle lengthening contractions (MLC). Representative western blot of phospho-Jak2 and Jak2 (4a) and phospho-Stat3 and Stat3 (4b). Graphs depict the ratio of phosphorylated to total protein. Data are presented as mean±SEM. Mean values represent the mean for all 8 subjects per time-point (8 samples per time-point, 40 samples total). (4c) Triple-immunofluorescent staining of 7 µm muscle cross-sections for satellite cells (green = Pax7^+^), phosphorylated STAT3 protein (red) and nuclei (DAPI = blue). White arrows denotes Pax7^+^/p-STAT3^+^ cell at 4 hr (T4), scale bar = 10 µm. Note: No p-STAT3 staining found at T0, T24, T72 or T120 (not shown)

It has been shown that IL-6-mediated muscle hypertrophy acts via the Janus kinase/signal transducers and activators of transcription (JAK/STAT3) signaling pathway [17]. To test if IL-6 was associated with JAK/STAT3 signaling in response to our MLC protocol, we analyzed JAK2 and STAT3 protein. Total and phosphorylated JAK2 and STAT3 were unchanged across all time-points following muscle damage (Fig. 4a, b). However, immunofluorescent staining revealed phosphorylated STAT3 (p-STAT3) co-localized with Pax7^+^ cells only at T4 in all subjects (Fig. 4c), with no detectable p-STAT3 at PRE, T24, T72 and T120 indicating that STAT3 signaling was transiently active within SCs at T4 (see Fig. S4).

**Figure 5:**
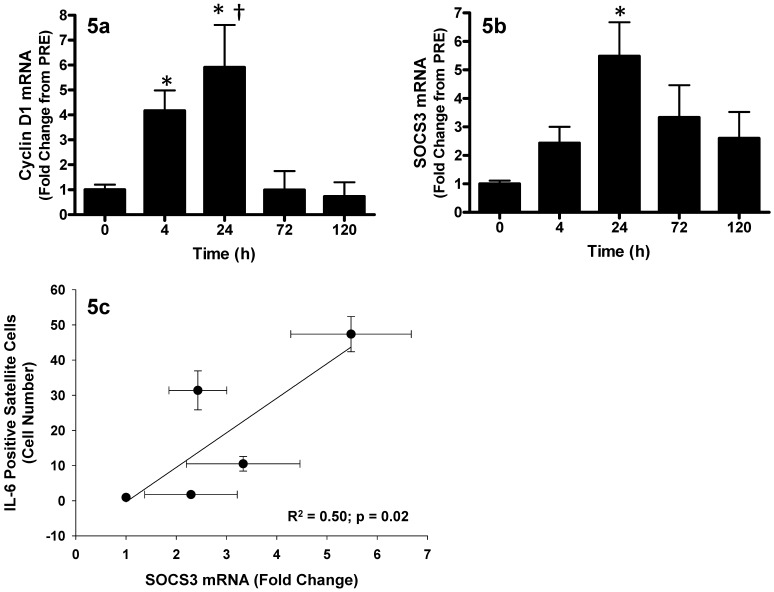
The response of downstream genes of STAT3 signaling following muscle lengthening contractions (MLC). (5a) Relative *cyclin D1* mRNA expression (fold-change from 0 hr). (5b) Relative *SOCS3* mRNA expression (fold-change from 0 hr). Values are reported as mean±S.E.M. (5c) Pearson correlation of IL-6^+^ satellite cells (number of cells) versus muscle *SOCS3* mRNA (fold-change), correlation is representative of the individual data points and presented as mean values (•)±SD (error bars). Mean values represent the mean for all 8 subjects per time-point (8 samples per time-point, 40 samples total). *p<0.05 vs. 0 hr; †p<0.05 vs. 4 hr.

In support of the observed p-STAT3 protein in SCs at T4, we observed increases in both *cyclin D1* and suppressor of cytokine signaling-3 (*SOCS3*) mRNA in whole muscle, which are both downstream genes regulated by IL-6-mediated JAK2/STAT3 signaling [35], [36]. *Cyclin D1* increased 4-fold at T4, peaked at T24 with a 6-fold increase in expression, and returned to baseline by T72 as compared to PRE (p<0.05; Fig. 5a). *SOCS3* mRNA expression increased 6-fold at T24 (P<0.05) and showed a trend to remain elevated at T72 (p = 0.07) returning to baseline by T120 (Fig. 5b). Furthermore, *SOCS3* mRNA was positively correlated with the number of IL-6^+^ SCs (R^2^ = 0.50, p = 0.02; Fig. 5c).

## Discussion

The release of growth factors and cytokines in response to muscle damage are an essential component of the muscle repair process [12], [16], [31], [37]–[39]. We have discovered that the pleiotropic effects of IL-6 extend to the human satellite cell (SC) compartment in response to eccentrically biased contraction-induced muscle damage. Importantly, we have shown that human muscle SCs express IL-6Rα in response to acute muscle injury suggesting that IL-6 may play a role in regulating SCs. In addition, we have shown that IL-6 is intimately associated with the SC response by a reporting a significant increase in IL-6 protein content within SCs at T4 and T24, coinciding with an increase in Pax7^+^ cell number at T24 and an increase in the percentage of Pax7^+^ cells actively proliferating (evidenced by an increased percentage Pax7^+^ cells expressing PCNA). We observed p-STAT3 within the SCs at T4, with the downstream genes *cyclin D1* and *SOCS3* increasing from T4-T24, suggesting that the IL-6/STAT3 signaling pathway was active within SCs early, during early proliferation, prior to the expansion of the SC population. These findings establish evidence that, in humans, IL-6 is fundamentally important to the contribution of SCs to muscle repair and may, at least in part, contribute to the proliferative component of the myogenic program [17], [29], [40].

We observed a transient increase in *IL-6* mRNA and IL-6 protein which agrees with previous findings where IL-6 peaked early after intense resistance exercise in humans or compensatory hypertrophy in animals [17], [41]. It is well established that IL-6 can be derived from skeletal muscle and released into circulation following acute exercise [25], [26], [29]. Thus, it is not surprising that our MLC protocol induced an increase in *IL-6* mRNA and circulating IL-6. In the present study, the increase in serum IL-6 was similar to previous studies which employed knee extensor or short duration exercise [42]–[45]. However, since the MLC protocol used in the present investigation only required approximately 90 seconds of time under tension, coupled with the relatively small muscle mass required by our protocol (*quadriceps* group only), the contribution of circulating IL-6 from the muscle was not as large as observed in studies involving strenuous whole-body exercise (i.e. 2-fold vs. 128-fold increase) [46]. In the present study a positive correlation between circulating IL-6 and muscle *IL-6* mRNA expression was observed (Fig. 1c), suggesting that muscle-derived IL-6 protein may be released into circulation accounting for the rise in plasma IL-6.

There are several mechanisms responsible for contraction-induced muscle IL-6 production. For example, an increase in intracellular calcium concentration in C2C12 myoblasts has been shown to be a potent activator of *IL-6* expression [29]. Although calcium concentrations were not measured in this study, it is possible that changes in intracellular calcium concentration as a result of repeated muscle contractions may have contributed to the increase in *IL-6* expression [27], [47]. In support of this notion it has previously been reported that calcium channel blockers administered prior to the same protocol used in the present investigation resulted in reduced skeletal muscle damage, suggesting a role for calcium-induced cellular damage [32]. Importantly, contraction-induced calcineurin-NFAT, IL-1, AP-1, NFκB, and nitric oxide signaling have been shown to induce *IL-6* gene expression in muscle [48], [49] (for an extensive review see ref. [26]), suggesting a potential role for calcium-induced IL-6 expression. Muscle produced IL-6 can act in an autocrine fashion, binding to the IL-6Rα on the sarcolemma, initiating the activation of the JAK/STAT3 signaling pathway [17], [26], [50]. Although it has been established that muscle can produce IL-6, SC-mediated IL-6 production has never been reported following injury in human tissue.

Satellite cells contained virtually no IL-6 protein prior to the MLC protocol (Fig. 3c). However, by T24, approximately 80% of all Pax7^+^ SCs were positive for IL-6. Interestingly, there was a positive correlation between the increase in IL-6 and the increase in PCNA co-localization with Pax7 early in the post-intervention time-course. Furthermore, the number of SCs was not significantly increased until T24, which suggests a potential role for IL-6 in newly proliferating SCs. The number of SCs peaked at T72, at which time IL-6^+^ SCs had decreased close to baseline levels (<10% IL-6^+^ cells). Interestingly, we have previously reported that *Myogenin* and *MRF4* mRNA peak at the same time-point that SC number peaks [39]. Since IL-6 was rapidly down-regulated prior to this time it is worth suggesting that IL-6 may be intimately involved in the proliferative process and the absence if IL-6 may be necessary for differentiation to occur. Additionally, the timing of increased IL-6 levels in the SCs in the current study is in agreement with previous *in vitro* findings from animal studies [51] that demonstrate a role for IL-6 in myoblast proliferation. In order to investigate this notion, *in vivo*, we examined JAK2/STAT3 signaling in muscle.

Upon IL-6 binding to the IL-6Rα, JAK2 is phosphorylated and initiates the phosphorylation of STAT3. STAT3 then forms a dimer and translocates to the nucleus where it can act as a transcription factor for several target genes [50]. JAK2/STAT3 signaling has been shown to induce the transcription of genes that regulate cell cycle progression and proliferation such as *cyclin D1* and *c-myc* [52]–[54]. In this study, there was no increase in whole muscle p-JAK2 or p-STAT3 protein at the time-points in which the muscle biopsies were obtained; however, the phosphorylation of STAT3 is very rapid and transient and it is possible that any increase in p-STAT3 detectable at the whole-muscle level occurred before T4 [41], [55]. Trenerry et al. (2007), observed an increase in p-STAT3 protein in whole muscle 2 h after intense resistance exercise that returned to baseline by 4 h. Alternatively, it is possible that changes in protein phosphorylation events within the SCs were too subtle to detect at the whole muscle level using standard western blotting techniques. Indeed, using immunofluorescent staining we showed p-STAT3 protein in the nuclei of SCs at T4 but no other time-point. The co-localization of p-STAT3 with Pax7 may be due to the phosphorylation of STAT3 protein within the SCs induced by the increase in IL-6 protein observed in the SCs at T4. IL-6 may act in an autocrine manner, binding to the IL-6Rα on the SC membrane, inducing the phosphorylation of STAT3 via GP130/JAK2 activation within the SC [17], [50], [55]. The timing of the appearance of p-STAT3 in the SCs is in agreement with observations made in rats demonstrating that SCs expressed phosphorylated STAT3 3 h following injury [55]. The authors of that study concluded that STAT3 signaling in SCs following muscle injury was important for successful muscle regeneration [55]. It is not surprising that p-STAT3 was found in the nuclei of SCs following muscle injury, as SCs represent the only resident mitotic cells within skeletal muscle and thus STAT3 signaling within these cells is inherently important for promoting the activation of genes such as *cyclin D1* which may help direct cell cycle progression and proliferation of the SC pool.

Our data demonstrate the timing of *cyclin D1* mRNA expression not only mirrors the protein expression of IL-6 in the satellite cells, but also coincides with the increasing proliferation of Pax7^+^ cells. Interestingly, IL-6^−/−^ mice demonstrated a significantly blunted number of p-STAT3^+^ SCs accompanied by a significantly lower level of *cyclin D1* expression after 3 d of compensatory hypertrophy as compared to wild-type animals [17]. In that study, the IL-6^−/−^ animals had less muscle hypertrophy and less SC-mediated myonuclear addition following compensatory overload, as a result of impaired SC proliferation [17]. These findings illustrate the importance of IL-6 mediated SC proliferation to the process of skeletal muscle growth following overload or injury.

In addition, the rapid down-regulation of *cyclin D1* at T72 illustrates the tight regulation of SC proliferation, and that rapid down-regulation of cell-cycle genes may be necessary for the induction of differentiation. In addition to inducing entry into the cell cycle, cyclin D1 may also act in a negative feedback manner to repress STAT3 activity by reducing nuclear p-STAT3 [56]. The reduction of nuclear p-STAT3 may be necessary for the induction of differentiation [57] and the timing of withdrawal of SC p-STAT3 observed in the present study (i.e. no p-STAT3 evident at T24 or later) supports this idea. Thus, the withdrawal of IL-6 mediated STAT3 signaling may be necessary for differentiation to occur. This notion is strengthened by our observations that IL-6 protein is evident in 80% of satellite cells at T24 and less than 10% of satellite cells at T72 corresponding to increases in *MRF4* and *Myogenin* (myogenic regulatory factors responsible for the initiation of differentiation) observed at T72 previously [39]. This provides further evidence that IL-6 signaling is withdrawn as differentiation begins.

STAT3 phosphorylation is regulated by SOCS3 as part of a negative feedback loop controlling the activation status of STAT3 proteins [58], [59]. SOCS3 can bind phosphotyrosines on the JAK2 receptors, physically blocking STAT3 binding, as well as biding to JAK2, directly blocking JAK kinase activity [50]. In addition, SOCS3 can also recruit ubiquitin-transferases, facilitating the ubiquitination of JAK2, targeting the JAK protein for proteosomal degradation [26], [50]. The expression of *SOCS3* positively correlated with the increase in IL-6^+^ SC, such that the subjects with the highest number of IL-6^+^ satellite cells had the highest expression of *SOCS3* mRNA (Fig. 5c). This correlation provides further support that IL-6 is signaling through the STAT3 pathway. Moreover, the timing of peak *SOCS3* expression coincides with the peak in satellite cell number and the down-regulation of *cyclin D1* expression. Trenerry et al. (2007), reported similar findings indicating that *SOCS3* mRNA expression peaked 2 h after intense resistance exercise, coinciding with the peak in *c-myc* mRNA and p-STAT3 protein, after which p-STAT3 signaling was no longer evident. The expression of *SOCS3* and the absence of other components of the IL-6/STAT3 signaling network beyond T24 illustrate the role of this network in promoting SC proliferation. In addition, the transient nature of IL-6 expression may be of critical importance in distinguishing IL-6 as a promoter of SC-mediated muscle repair.

Classically, elevated IL-6 has been implicated in muscle wasting diseases such as cachexia [23]. The pleiotropic roles of IL-6 appear to be dependent on the stimuli, nature of the immune and local tissue responses, and the temporal expression of IL-6 in inducing different phenotypic traits [17], [21], [23]. This study examined IL-6 signaling in the context of muscle stem cell proliferation induced by MLC. Our findings compliment recent data that implicate IL-6 as an essential regulator of SC-mediated hypertrophy in mice and provide insight into the control of the SC response to muscle injury and repair in humans. In summary, these findings suggest that IL-6 may play a key role in SC proliferation in humans by inducing genes such as *cyclin D1* through the activation of JAK2/STAT3 signaling and that IL-6 is a factor mediating the repair response to acute muscle damage.

## Materials and Methods

### Subjects

Eight healthy males (age 22±1 y, height 185±2 cm, weight 79±8 kg) were recruited from the McMaster University community. Subjects underwent a routine medical screening, completed a health questionnaire, and were required to not have been involved in a lower-body resistance exercise training program for at least 6 months prior to participating in the study. Subjects were told to refrain from exercising throughout the time-course of the study. All subjects were informed of the procedures and potential risks associated with the study and gave their written informed consent to participate. This study was approved by the Hamilton Health Sciences Research Ethics Board and conformed to all declarations on the use of human subjects as research participants.

### Muscle Damage Protocol

We employed a protocol involving maximal isokinetic unilateral muscle lengthening contractions of the *quadriceps femoris* performed on a Biodex dynamometer (Biodex-System 3, Biodex Medical Systems, Inc., USA) at 3.14 rad.s^−1^. Briefly, for each subject, one leg was selected randomly to complete the protocol described below. Movement at the shoulders, hips, and thigh were restrained with straps in order to isolate the knee extensors during the protocol.

Immediately prior to the intervention, subjects underwent a brief familiarization trial involving 5 to 10 submaximal lengthening contractions of the leg under investigation. Subjects were required to perform 30 sets of 10 maximal muscle lengthening contractions with one minute rest between sets, for a total of 300 lengthening contractions. During each set, investigators provided verbal encouragement in an attempt to elicit a maximal effort. It has been well documented that our protocol induces a significant level of skeletal muscle damage and substantial cellular disruption evidenced by extensive z-band streaming, desmin disruption, a significant increase in plasma creatine kinase (3.9-fold increase 48 h after exercise), a significant infiltration of macrophages and neutrophils (4-fold and 14- fold increase 48 h after exercise respectively) in addition to a significant myogenic regulatory factor and satellite cell response (based on NCAM staining) [12], [32]–[34], [39].

### Muscle Biopsies

Five percutaneous needle biopsies were obtained from the mid-portion of the *vastus lateralis* under local anesthetic (1% lidocaine) using manual suction [60], [61]. One muscle biopsy was obtained from the non-working leg prior to the intervention. Baseline measures were generated from the pre-intervention biopsy (PRE) taken prior to beginning the damage protocol. Four biopsies were obtained from the working leg at different time points following the intervention. Muscle biopsies and blood draws were taken concurrently, at 4 h (T4), 24 h (T24), 72 h (T72), and 120 h (T120) post-intervention each from a new incision 3–5 cm proximal to the last biopsy site. Approximately 150 mg of muscle tissue was collected from each biopsy. Following collection of the sample, the muscle was dissected free of adipose and connective tissue, divided into separate pieces for RNA and protein analysis, flash-frozen in liquid nitrogen, and stored at −80°C for later analysis. Approximately 25 mg of each sample was mounted in Optimum Cutting Temperature (OCT) compound and frozen in isopentane cooled in liquid nitrogen. For each subject, 4, 24, 72 and 120 h biopsies were conducted at approximately the same time of day (±1 hour) in order to minimize variability between subjects.

### Immunofluorescence

7 µm sections were cryosectioned and stained with antibodies against Pax7 (neat; DSHB, USA); IL-6 (500 ng/mL, MAB 2061, R&D Systems, USA); p-STAT3 (p-STAT3 Y705 1∶100, Cell Signaling Technologies Inc., USA); IL-6Rα (1∶50, MCA822, Serotec, UK); PCNA (1∶200, ab15497, Abcam Inc., USA); and Laminin (1∶1000, L8271, Sigma-Aldrich, Canada). Secondary antibodies used were: Pax7 (AlexaFluor 488 or AlexaFluor 594, 1∶500, Invitrogen, Molecular Probes Inc., USA); IL-6, IL-6Rα (immunoglobulin biotinylated secondary antibody, 1∶200, Dako Canada, Inc.; followed by a streptavidin-FITC fluorochrome, 1∶100, Biosource. USA); p-STAT3 (R-Phycoerythrin; 1∶200; Pierce, USA); PCNA (Texas Red, 1∶500, Invitrogen, Molecular Probes Inc., USA) and Laminin (AlexaFluor 488, 1∶500, Invitrogen, Molecular Probes Inc., USA). For co-immunofluorescent staining for Pax7 and IL-6, Pax7 and IL-6Rα, Pax7 and Laminin, and Pax7 and p-STAT3 sections were fixed with 2% paraformaldehyde (PFA, Sigma, USA) for 10 min followed by several washes in PBS. Sections were then covered for 30 min in a blocking solution containing, 2% BSA, 5% FBS, 0.2% Triton-X 100, 0.1% sodium azide; whereas for Pax7 and PCNA co-staining, fixing was done with ice-cold acetone, and blocked with 10% goat serum (GS) in 0.01% Triton-X 100 (Sigma, USA). Following blocking, sections were incubated in the primary antibody at 4°C overnight. After several washes, sections were then incubated in the appropriate secondary antibodies. Sections were then re-fixed in 2% PFA (Sigma, USA) to prevent migration of the secondary antibodies and re-blocked in 10% GS in 0.01% Triton-X 100 (Sigma, USA). The sections were then incubated in the second primary antibody, followed by incubation in the appropriate secondary antibody. Sections were then washed with PBS and 4′,6-diamidino-2-phenylindole (DAPI) (Sigma, USA) for nuclear staining. Staining was verified using the appropriate positive and negative controls to ensure specificity of staining (see Fig. S5). Stained slides were viewed with the Nikon Eclipse 90*i* Microscope (Nikon Instruments, Inc., USA) and images were captured and analyzed using the Nikon NIS Elements 3.0 software (Nikon Instruments, Inc., USA).

### Immunofluorescent Analysis

All immunostaining quantification and enumerations were conducted on all subjects at all time points (n = 8, 5 time-points, 40 biopsies total). For each time-point, images were taken at 40× to ensure a high resolution image to accurately determine nuclei which were associated with myofibers and displayed co-localization of immunolabeling. For each time-point measurement at least 15 different images per subject, per time-point were taken corresponding to at least 1000 myonuclei.

#### Satellite cell enumeration

Satellite cell enumeration was conducted with an anti-Pax7 antibody staining satellite cells and DAPI staining all nuclei in the muscle cross sections. Only nuclei which stained positive for Pax7 and DAPI were counted as satellite cells. In order to minimize enumeration error only nuclei associated with myofibers were counted and interstitial nuclei (based on their location relative to the basal lamina) were excluded from enumeration. Data are presented with Pax7 positive (Pax7^+^) cells as a percentage of total myonuclei.

#### IL-6 positive satellite cells enumeration

Enumeration was conducted using triple-immunolabeling with antibodies against IL-6 and Pax7 and using DAPI to stain nuclei. Only nuclei associated with myofibers were used to enumerate the percentage of Pax7^+^ cells which stained positive for IL-6 (Pax7^+^/IL-6^+^). Data are presented with IL-6+ satellite cells (Pax7^+^/IL-6^+^) as a percentage of total Pax7^+^ cells as well as a percentage of total myonuclei.

#### Proliferating cell nuclear antigen (PCNA) positive satellite cells

Enumeration was conducted using triple-immunolabeling with antibodies against PCNA and Pax7 and using DAPI to stain nuclei. Only nuclei associated with myofibers were used to enumerate the percentage of Pax7^+^ cells which stained positive for PCNA (Pax7^+^/PCNA^+^). Data are presented with PCNA^+^ satellite cells (Pax7^+^/PCNA^+^) as a percentage of total Pax7^+^ cells.

### Blood Measures

A resting blood sample was obtained from the antecubital vein immediately prior to the intervention. Blood was also drawn at T4, T24, T72 and T120. Approximately 5 mL of blood was collected and separated into one heparinized and one non-heparinized vacutainer tube at each time-point. Samples were separated into 50 µL aliquots and stored at −80°C for analysis at a later date. Serum samples were thawed on ice and analyzed for IL-6 protein using a commercially available Enzyme-Linked ImmunoSorbant Assay (ELISA) kit according to manufactures instructions (R&D Systems, Inc., USA). Samples were run in duplicate with an average intra-assay CV of less than 2% with all subjects run on the same plate.

### RNA isolation

RNA was isolated from homogenized muscle samples using the TRIzol/RNeasy method. Briefly, approximately 25 mg of each muscle sample was homogenized in a total of 1.0 mL of TRIzol Reagent (Invitrogen Corporation, Canada) using a rotary homogenizer. Homogenized samples were incubated at room temperature for 5 min followed by the addition of 0.2 mL of chloroform then shaken vigorously for 15 s. After another 5 min incubation at room temperature, samples were centrifuged at 12 000 g at 4°C for 10 min. The aqueous phase was then transferred to a new tube and the volume was measured. 1 volume of 70% ethanol was added to the aqueous phase and mixed. Multiple 700 µL aliquots were then transferred into a Qiagen RNeasy mini spin column and RNA was purified by using the RNeasy mini kit (Cat. # 74106), following the manufacturer's instructions (Qiagen Sciences, USA). The RNA was quantified and purity was assessed using a spectrophotometer (NanoDrop 1000, Thermo Scientific, USA). Random samples were also tested for RNA quality using denaturing gel electrophoresis.

### Reverse Transcription (RT)

Individual samples were reverse transcribed in 20 µL reactions using a commercially available kit (Applied Biosystems High Capacity cDNA Reverse Transcription Kit; Applied Biosystems, USA) according to the manufacturer's instructions. The cDNA synthesis reaction was carried out using an Eppendorf Mastercycle ep*gradient* thermal cycler (Eppendorf, Canada).

### Quantitative Real-Time Polymerase Chain-Reaction (qRT-PCR)

Individual 25 µL reactions were prepared in 0.2 mL Stratagene PCR tubes (Stratagene, USA) and run in duplicate for each time-point. Primers were custom-made using published sequences (Table 1) and were re-suspended in 1X TE buffer (10 mM Tris-HCl, 0.11 mM EDTA) and stored at −20°C prior to use. In each reaction tube, 1.0 µL of cDNA and 7.5 µL of ddH_2_O were added to 16.5 µL of a master mix containing 12.5 µL of RT^2^ Real-Time SYBR Green/Rox PCR master mix (SuperArray Bioscience Corp., USA) along with 2 µL of the specific forward and reverse primers. qRT-PCR reactions were carried out using a Stratagene Mx3000P real-time PCR System (Stratagene, USA) using Stratagene MxPro QPCR Software Version 3.00 (Stratagene, USA). Fold changes in gene expression were calculated using the delta-delta Ct method [62] normalized to the housekeeping gene *glyceraldehyde 3-phosphate dehydrogenase (GAPDH)* (Table 1). Thus, mRNA values were expressed as a fold change from PRE (mean±SEM). GAPDH expression was not different from PRE at any of the post-intervention time-points.

**Table 1:** qRT-PCR Primer Sequences.

Gene Name	Forward Sequence	Reverse Sequence	Entrez Gene Accession Number
*IL-6*	5′-GAAAGCAGCAAAGAGGCACT-3′	5′-AGCTCTGGCTTGTTCCTCAC-3′	3569
*IL-6Rα*	5′-GACAATGCCACTGTTCACTG-3′	5′GCTAACTGGCAGGAGAACTT-3′	3570
*Cyclin D1*	5′-CACGATTTCATTGAACACTTCC-3′	5′-TGAACTTCACATCTGTGGCAC-3′	595
*SOCS3*	5′-GACCAGCGCCACTTCTTCA-3′	5′-CTGGATGCGCAGGTTCTTG-3′	9021
*GAPDH*	5′-CCTCCTGCACCACCAACTGCTT-3′	5′-GAGGGGCCATCCACAGTCTTCT-3′	2597

*IL-6*, interleukin-6; *IL-6Rα*, interleukin-6 receptor; *SOCS3*, suppressor of cytokine signaling 3; *GAPDH*, glyceraldehyde 3-phosphate dehydrogenase.

### Western Blot Analysis

Muscle homogenates (7% wt/v) were prepared in ice-cold homogenization buffer (20 mM Tris-HCl, 1 mM Na_3_VO_4_, 50 mM NaF, 40 mM β-glycerolphosphate, 20 mM NaPyrphosphate, 0.5% Triton-X-100, 2 complete mini Roche protease inhibitor tabs, pH 7.2). Equal amounts (50 µg) of protein, as determined by Bradford Assay (Thermo Fisher Scientific, USA), were loaded into lanes of a 7.5% gel, separated at 100 V for 90 min then transferred onto a polyvinyl difluoride (PVDF) membrane (Millipore, Canada) for 60 min at 70 V. After blocking for 1 h at 4°C in 5% bovine serum albumin (BSA, Santa Cruz Biotechnology, USA) blots were probed with phospho-specific primary antibody overnight at 4°C then relevant secondary antibody. After phosphorylated protein detection by ECL (SuperSignal West Dura; Thermo Fisher Scientific, USA) and Alpha Innotech FluorChem SP (Alpha Innotech Corporation, USA) membranes were stripped with Restore Western Blot Stripping Buffer (Thermo Fisher Scientific, USA) for 20 min at room temperature and detection of total-specific antibodies was conducted. Bands were quantified using AlphaEase FC Software, Version 5.0.2 (Alpha Innotech Corporation, USA). Levels of phosphorylated protein were expressed relative to total.

The following primary antibodies were used; phospho-Stat3 (Tyr705) 1∶1000, phospho-Jak2 (Tyr1007/1008) 1∶500, Stat3 1∶1000, and Jak2 (D2E12)1∶500. All primary antibodies were raised in rabbit and purchased from Cell Signaling Technology, USA. A goat polyclonal to Rabbit IgG (HRP) secondary antibody (1∶50,000; Abcam Inc., USA) was used.

### Statistical Analysis

Statistical analysis was performed using SigmaStat 3.1.0 analysis software (Systat, SPSS Inc., USA). Serum IL-6 concentrations, mRNA, protein, Pax7^+^/IL-6^+^, and Pax7^+^/PCNA^+^ enumeration were analyzed using a 1-way repeated-measures analysis of variance (ANOVA). Correlations were analyzed using the Pearson Product Moment correlation. Statistical significance was accepted at *P*<0.05. Significant interactions and main effects were analyzed using the Tukey's HSD post hoc test. All results are presented as means±SEM.

## Supporting Information

Figure S1 Muscle satellite (stem) cell response to muscle-lengthening contractions (MLC): Triple-immunofluorescent staining of 7 µm muscle cross-section for satellite cells (red = Pax7+), laminin (green) and nuclei (DAPI = blue). Thin arrows denote the Pax7+ cells located beneath the basal lamina in the satellite cell niche (20× objective). (S1a): Pre-intervention; (S1b): 4 hours (T4); (S1c): 24 hours (T24); (S1d): 72 hours (T72); (S1e): 120 hours (T120) post-intervention.
(7.26 MB TIF)

Figure S2 Satellite cell IL-6 protein expression following muscle lengthening contractions (MLC): Triple-immunofluorescent staining of 7 µm muscle cross-sections for satellite cells (red = Pax7+), IL-6 protein (green) and nuclei (DAPI = blue). (Figures S2a–c: 20× objective; Figures S2d–f: 40× objective). (S2a): Pre-intervention (T0), note an absence of IL-6 co-localization (20×); (S2b): Higher magnification of T0 (40×) arrows denote Pax7+ cells with no IL-6 positivity; (S2c): 4 hours (T4), note some IL-6 positivity (20×) see manuscript for higher magnification image of T4; (S2d): 24 hours (T24), note increased IL-6 expression and Pax7/IL-6 co-localization (20×); (S2e): Higher magnification of T24 (40×) showing satellite cells staining positive for IL-6. (S2f): 120 hours (T120) post-intervention (40×), note an absence of IL-6 co-localization. Note for T72 see manuscript.
(9.94 MB TIF)

Figure S3 Satellite cell co-localization with proliferating cell nuclear antigen (PCNA) following muscle lengthening contractions (MLC): Triple-immunofluorescent staining of 7 µm muscle cross-section for satellite cells (green = Pax7+), PCNA (red) and nuclei (DAPI = blue). (S3a): Pre-intervention (T0), note absence of PCNA; (S3b): 4 hours (T4); (S3c): 24 hours (T24); (S3d): 72 hours (T72); (S3e): 120 hours (T120) post-intervention. All images acquired with 40× objective.
(9.12 MB TIF)

Figure S4 Satellite cell co-localization with phosphorylated STAT3 protein following muscle lengthening contractions (MLC): Triple-immunofluorescent staining of 7 µm muscle cross-sections for satellite cells (green = Pax7+), phosphorylated STAT3 protein (red) and nuclei (DAPI = blue). (S4a): Pre-intervention note absence of satellite cell associated p-STAT3; (S4b): 4 hours (T4); (S4c): 24 hours (T24), note absence of satellite cell associated p-STAT3. Images acquired with 40× objective.
(5.17 MB TIF)

Figure S5 Control Images: (S5a): Immunofluorescent (IF) stain of 7 µm muscle cross-sections for IL-6Rα (green - streptavidin-FITC), nuclei (DAPI) and the secondary antibody used for Pax7 (secondary only - Alexa 594) acquired using the 20× objective. Arrows denote nuclei that co-localize with IL-6Rα and do not show any non-specific secondary binding of the Alexa 594, or any Alexa 594 interaction with the IL-6Rα antibody. (S5b): Immunofluorescent (IF) stain of 7 µm muscle cross-sections for Pax7 (red - Alexa 594), nuclei (DAPI) and the secondary antibody used for IL-6 (secondary - tertiary only: immunoglobulin biotinylated secondary antibody + streptavidin-FITC) acquired using the 20× objective. Arrows denote nuclei that co-localize with Pax7 and do not show any non-specific secondary binding of the secondary antibodies (FITC), or any FITC interaction with the Pax7 antibody. (S5c): Immunofluorescent (IF) stain of 7 µm muscle cross-sections for IL-6 (green - streptavidin FITC), nuclei (DAPI) and the secondary antibody used for Pax7 (secondary only - Alexa 594) acquired using the 20× objective. Arrows denote nuclei that co-localize with IL-6 and do not show any non-specific secondary binding of the Alexa 594, or any Alexa 594 interaction with the IL-6 antibody. (S5d): Immunofluorescent (IF) stain of 7 µm muscle cross-sections for PCNA (red - Texas Red), nuclei (DAPI) and the secondary antibody used for Pax7 (secondary only - Alexa 488) acquired using the 20× objective. Arrows denote nuclei that co-localize with PCNA and do not show any non-specific secondary binding of the Alexa 488, or any Alexa 488 interaction with the PCNA antibody. (S5e): Immunofluorescent (IF) stain of 7 µm muscle cross-sections for Pax7 (green - Alexa 488), nuclei (DAPI) and the secondary antibody used for PCNA (secondary only: Texas Red) acquired using the 20× objective. Arrows denote nuclei that co-localize with Pax7 and do not show any non-specific secondary binding of the secondary antibody (Texas Red), or any Texas Red interaction with the Pax7 antibody. (S5f): Immunofluorescent (IF) stain of 7 µm muscle cross-sections for phosphorylated STAT3 (p-STAT3) (red - Texas Red), nuclei (DAPI) and the secondary antibody used for Pax7 (secondary only - Alexa 488) acquired using the 20× objective. Arrows denote nuclei that co-localize with p-STAT3 and do not show any non-specific secondary binding of the Alexa 488, or any Alexa 488 interaction with the PCNA antibody.
(9.86 MB TIF)
