# Supplementary figures and images for: Association of Interleukin-6 Signalling with the Muscle Stem Cell Response Following Muscle-Lengthening Contractions in Humans
Source: PLoS One. 2009 Jun 24;4(6):e6027. doi: 10.1371/journal.pone.0006027 (PMC2696599; doi:10.1371/journal.pone.0006027)

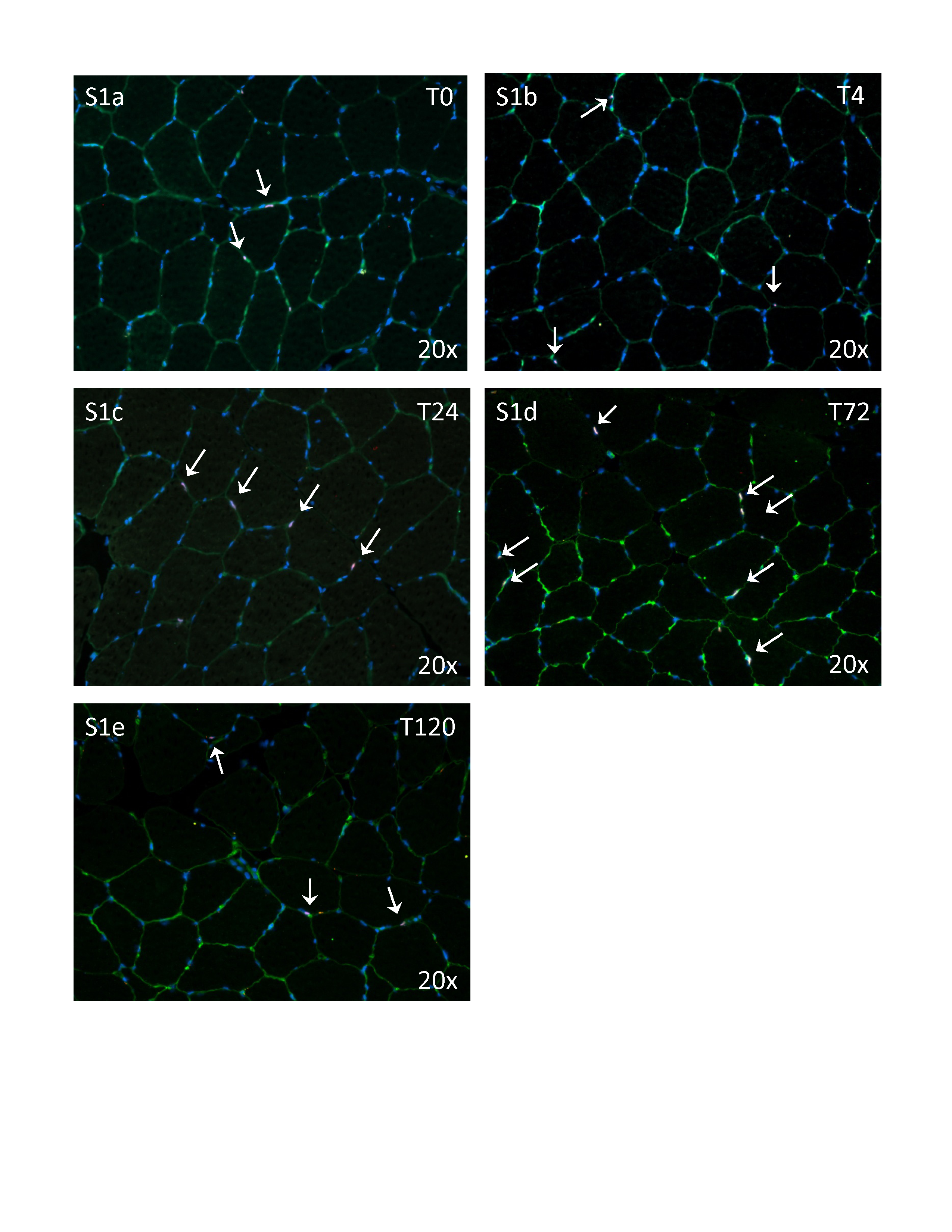

Supplement: Figure S1 — Muscle satellite (stem) cell response to muscle-lengthening contractions (MLC): Triple-immunofluorescent staining of 7 µm muscle cross-section for satellite cells (red = Pax7+), laminin (green) and nuclei (DAPI = blue). Thin arrows denote the Pax7+ cells located beneath the basal lamina in the satellite cell niche (20× objective). (S1a): Pre-intervention; (S1b): 4 hours (T4); (S1c): 24 hours (T24); (S1d): 72 hours (T72); (S1e): 120 hours (T120) post-intervention. (7.26 MB TIF) [file pone.0006027.s001.tif]

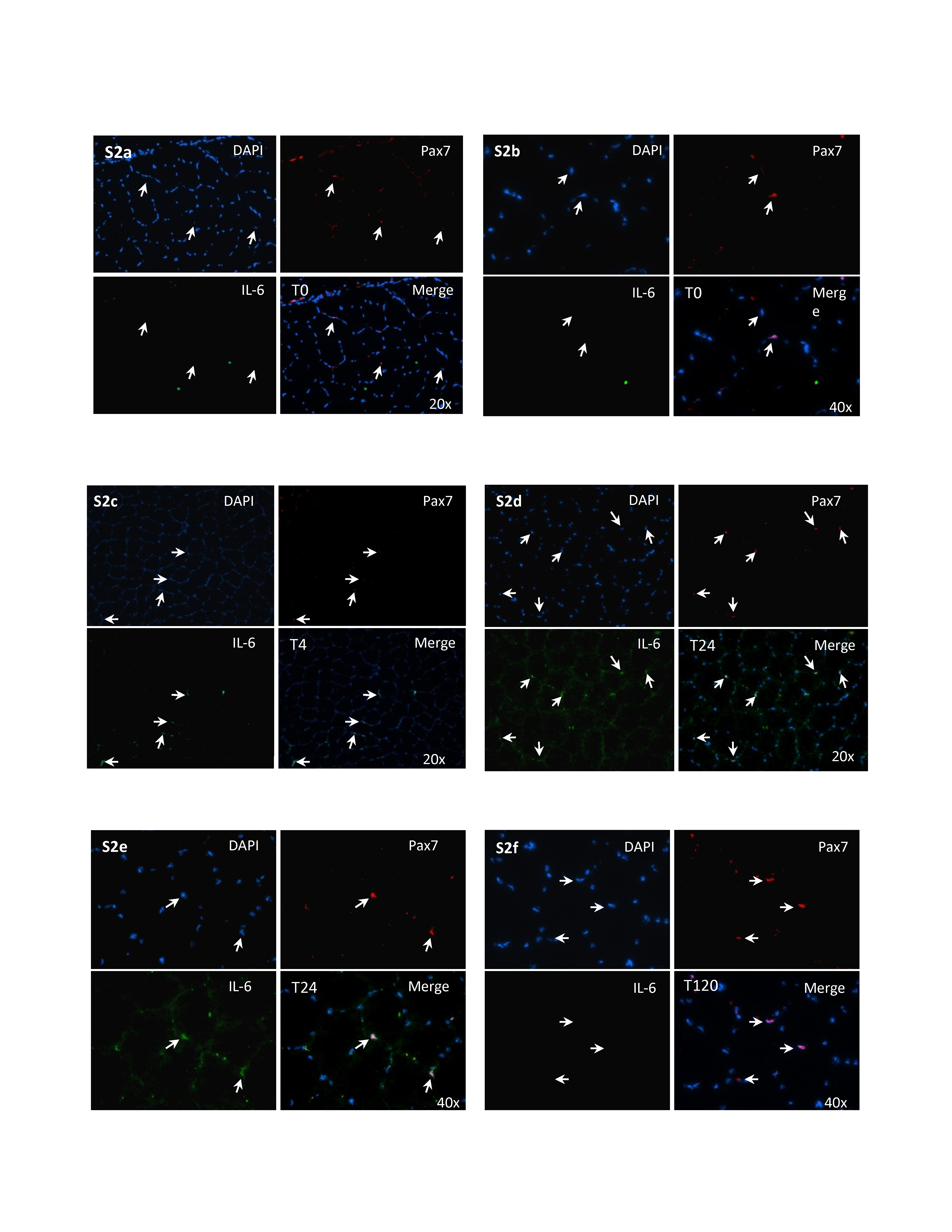

Supplement: Figure S2 — Satellite cell IL-6 protein expression following muscle lengthening contractions (MLC): Triple-immunofluorescent staining of 7 µm muscle cross-sections for satellite cells (red = Pax7+), IL-6 protein (green) and nuclei (DAPI = blue). (Figures S2a–c: 20× objective; Figures S2d–f: 40× objective). (S2a): Pre-intervention (T0), note an absence of IL-6 co-localization (20×); (S2b): Higher magnification of T0 (40×) arrows denote Pax7+ cells with no IL-6 positivity; (S2c): 4 hours (T4), note some IL-6 positivity (20×) see manuscript for higher magnification image of T4; (S2d): 24 hours (T24), note increased IL-6 expression and Pax7/IL-6 co-localization (20×); (S2e): Higher magnification of T24 (40×) showing satellite cells staining positive for IL-6. (S2f): 120 hours (T120) post-intervention (40×), note an absence of IL-6 co-localization. Note for T72 see manuscript. (9.94 MB TIF) [file pone.0006027.s002.tif]

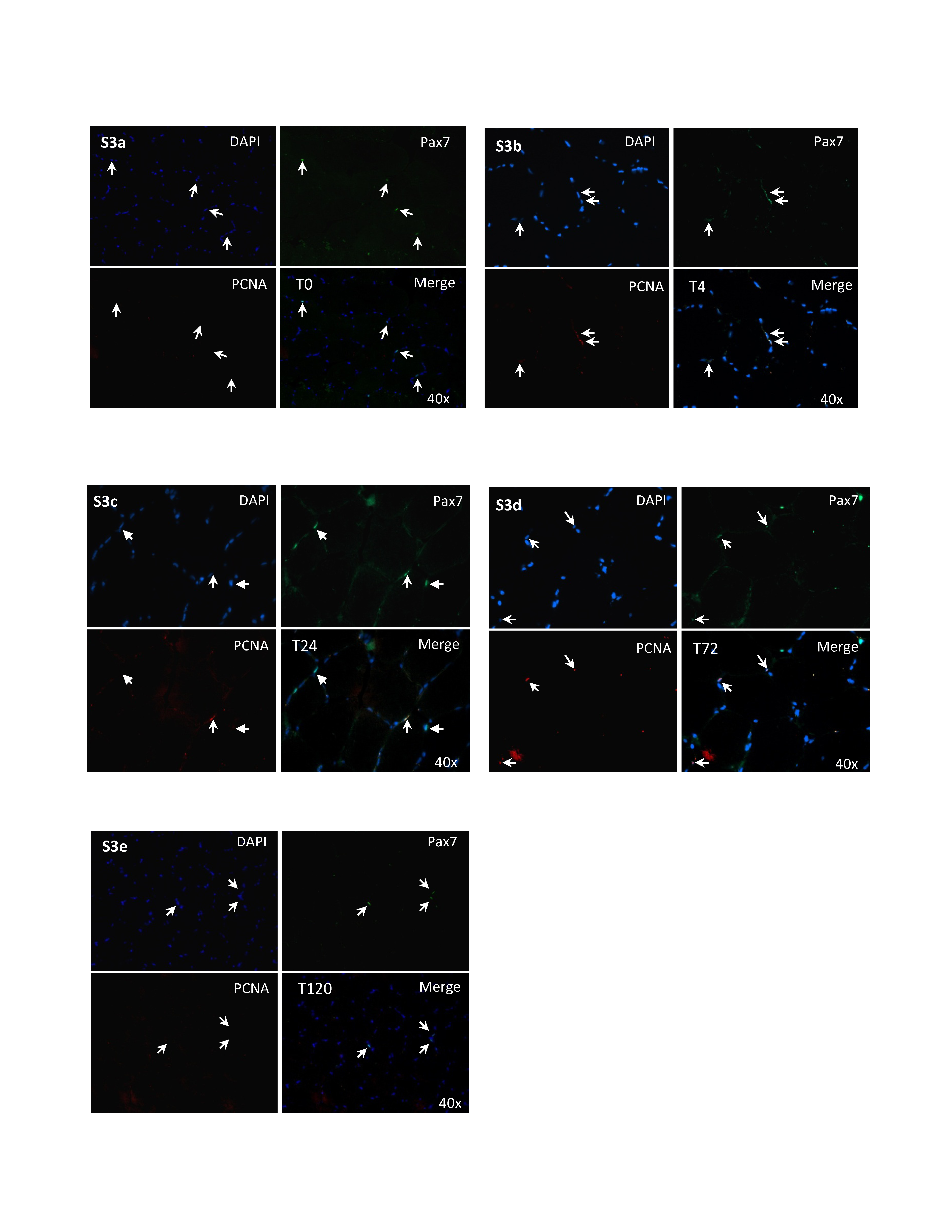

Supplement: Figure S3 — Satellite cell co-localization with proliferating cell nuclear antigen (PCNA) following muscle lengthening contractions (MLC): Triple-immunofluorescent staining of 7 µm muscle cross-section for satellite cells (green = Pax7+), PCNA (red) and nuclei (DAPI = blue). (S3a): Pre-intervention (T0), note absence of PCNA; (S3b): 4 hours (T4); (S3c): 24 hours (T24); (S3d): 72 hours (T72); (S3e): 120 hours (T120) post-intervention. All images acquired with 40× objective. (9.12 MB TIF) [file pone.0006027.s003.tif]

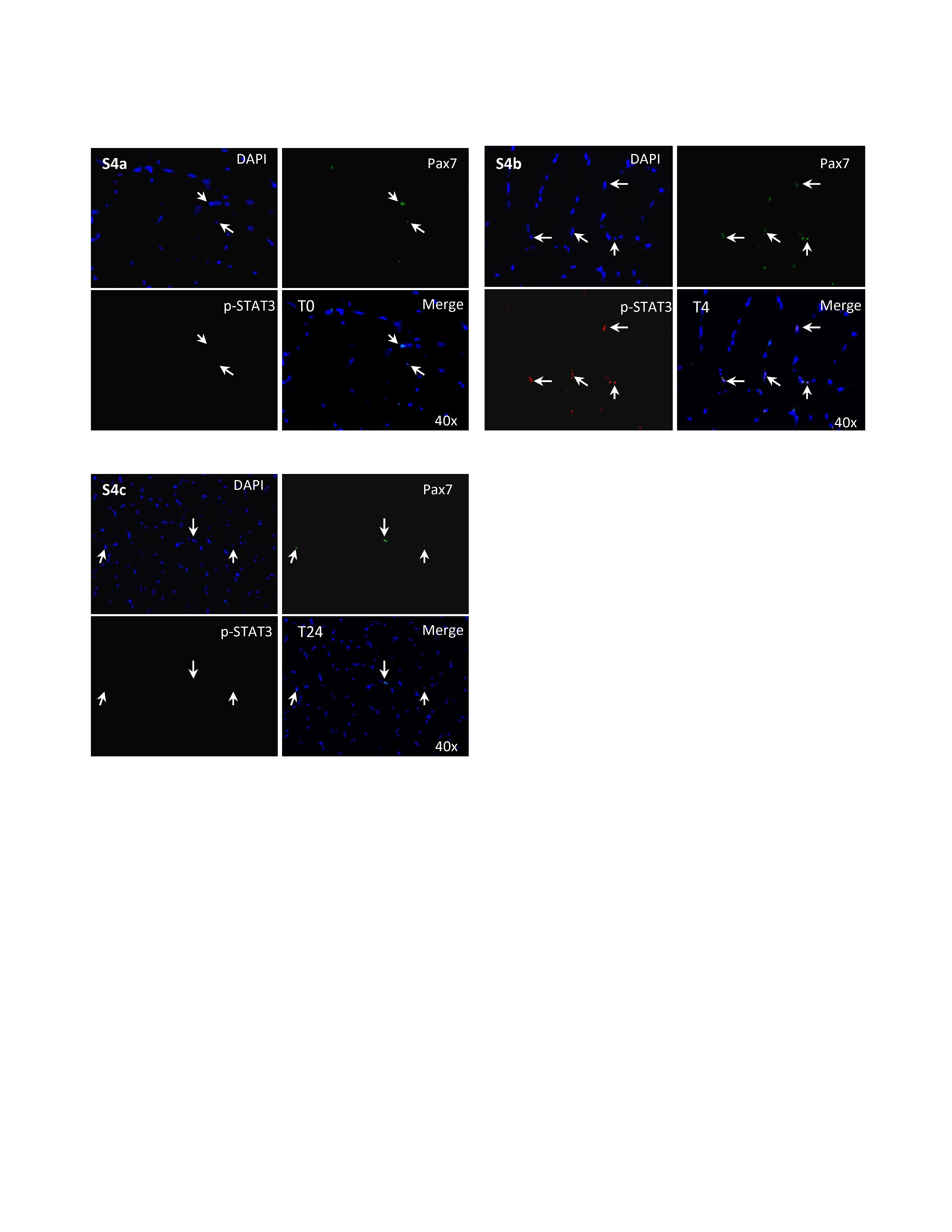

Supplement: Figure S4 — Satellite cell co-localization with phosphorylated STAT3 protein following muscle lengthening contractions (MLC): Triple-immunofluorescent staining of 7 µm muscle cross-sections for satellite cells (green = Pax7+), phosphorylated STAT3 protein (red) and nuclei (DAPI = blue). (S4a): Pre-intervention note absence of satellite cell associated p-STAT3; (S4b): 4 hours (T4); (S4c): 24 hours (T24), note absence of satellite cell associated p-STAT3. Images acquired with 40× objective. (5.17 MB TIF) [file pone.0006027.s004.tif]

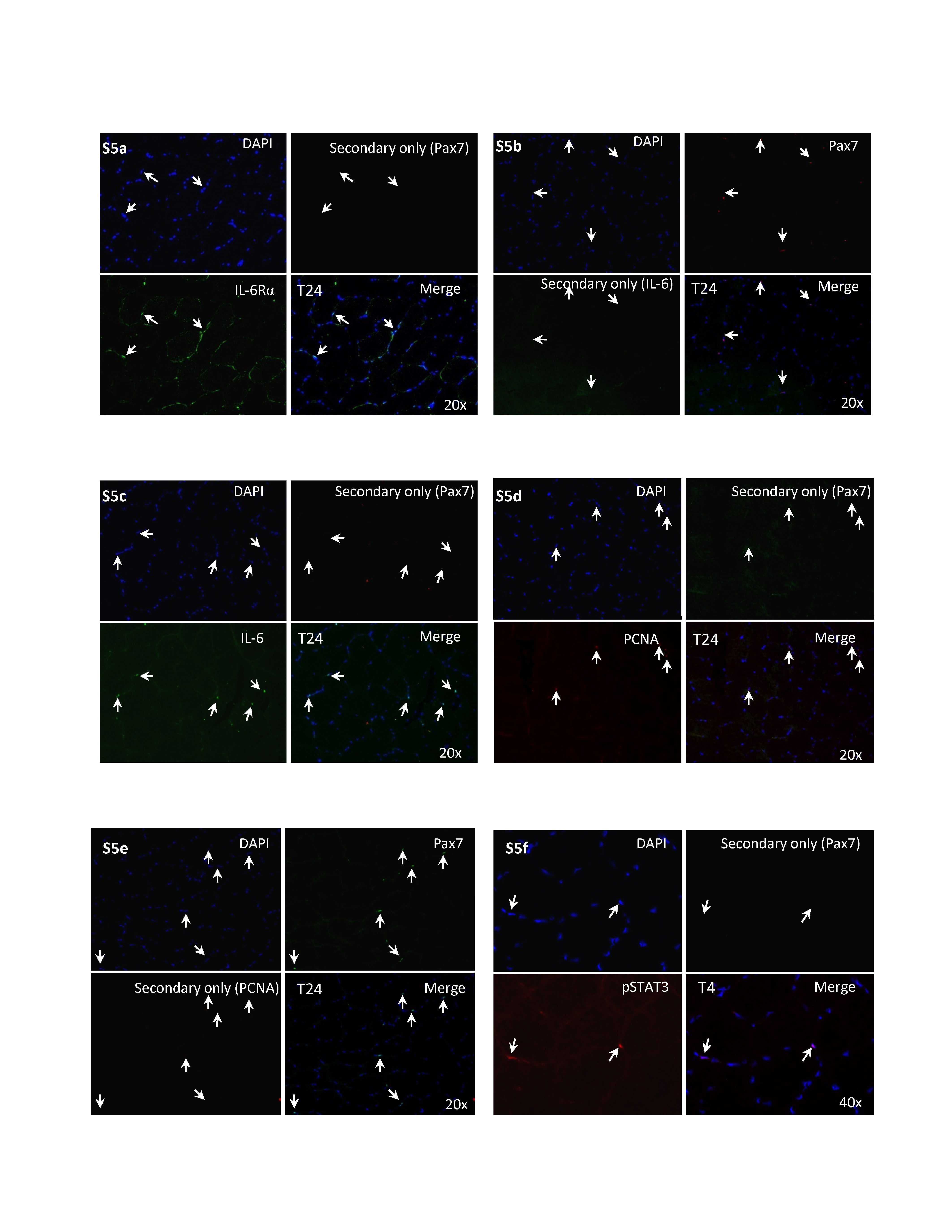

Supplement: Figure S5 — Control Images: (S5a): Immunofluorescent (IF) stain of 7 µm muscle cross-sections for IL-6Rα (green - streptavidin-FITC), nuclei (DAPI) and the secondary antibody used for Pax7 (secondary only - Alexa 594) acquired using the 20× objective. Arrows denote nuclei that co-localize with IL-6Rα and do not show any non-specific secondary binding of the Alexa 594, or any Alexa 594 interaction with the IL-6Rα antibody. (S5b): Immunofluorescent (IF) stain of 7 µm muscle cross-sections for Pax7 (red - Alexa 594), nuclei (DAPI) and the secondary antibody used for IL-6 (secondary - tertiary only: immunoglobulin biotinylated secondary antibody + streptavidin-FITC) acquired using the 20× objective. Arrows denote nuclei that co-localize with Pax7 and do not show any non-specific secondary binding of the secondary antibodies (FITC), or any FITC interaction with the Pax7 antibody. (S5c): Immunofluorescent (IF) stain of 7 µm muscle cross-sections for IL-6 (green - streptavidin FITC), nuclei (DAPI) and the secondary antibody used for Pax7 (secondary only - Alexa 594) acquired using the 20× objective. Arrows denote nuclei that co-localize with IL-6 and do not show any non-specific secondary binding of the Alexa 594, or any Alexa 594 interaction with the IL-6 antibody. (S5d): Immunofluorescent (IF) stain of 7 µm muscle cross-sections for PCNA (red - Texas Red), nuclei (DAPI) and the secondary antibody used for Pax7 (secondary only - Alexa 488) acquired using the 20× objective. Arrows denote nuclei that co-localize with PCNA and do not show any non-specific secondary binding of the Alexa 488, or any Alexa 488 interaction with the PCNA antibody. (S5e): Immunofluorescent (IF) stain of 7 µm muscle cross-sections for Pax7 (green - Alexa 488), nuclei (DAPI) and the secondary antibody used for PCNA (secondary only: Texas Red) acquired using the 20× objective. Arrows denote nuclei that co-localize with Pax7 and do not show any non-specific secondary binding of the secondary antibody (Texas Red), [file pone.0006027.s005.tif]
